# Editorial: Preventative medicine: nutritional and lifestyle interventions for healthy ageing and chronic diseases

**DOI:** 10.3389/fnut.2025.1723877

**Published:** 2025-12-04

**Authors:** Faith Kwa, Macarena Lozano-Lorca

**Affiliations:** 1 Department of Biomedical, Health and Exercise Sciences, School of Health Sciences, Swinburne University of Technology, Hawthorn, VIC, Australia; 2 Department of Preventive Medicine and Public Health, University of Granada, Granada, Spain; 3 Instituto de Investigación Biosanitaria ibs.GRANADA, Granada, Spain; 4 Centro de Investigación Biomédica en Red de Epidemiología y Salud Pública, Instituto de Salud Carlos III, Madrid, Spain

**Keywords:** lifestyle, diet, exercise, sleep, cancer, eye disorders, musculoskeletal diseases, metabolic disease

With the global aging population rising at an unprecedented rate, there is an urgent need for comprehensive strategies that support healthy aging and effectively manage chronic diseases (1). These efforts are critical to alleviating the substantial socio-economic and personal burdens associated with aging-related health challenges. While healthcare providers play a central role in guiding individuals toward healthier lifestyles through education and medical interventions, and community-driven support systems fostering environments that encourage and sustain health-promoting behaviors, the responsibility for healthy aging ultimately lies with individuals, who must cultivate habits of self-care and self-management from an early age (2). Key lifestyle interventions include sufficient sleep, regular physical activity, balanced nutrition, and the use of nutritional supplements where appropriate (3). A proactive focus on chronic disease prevention is also essential and evidence of lifestyle and nutritional interventions to enhance quality of life across the lifespan is presented here (4).

Cancer accounts for one in every six deaths experienced globally (5). Lung cancer, one of the most prevalent types affecting over 2 million people worldwide, leads in the death toll of people with cancer (5). Apart from the strong health advice for people to not engage in smoking-related activities, it is essential to scrutinize the various lifestyle interventions that can help to alleviate the risk of carcinogenesis in the lung (6). Shi et al. investigated if adhering to the 2015 Dutch Dietary Guidelines (i.e., more plant-based food intake and decreased consumption of meat-based products and sugar-based beverages) can reduce the incidence and mortality of different types of lung cancer (e.g., small cell lung cancer and non-small cell lung cancer). Over the course of the study, it was reported that 1,642 new cases were identified over 8.8 years and 1,172 lung cancer-related deaths were reported within a 15.1-year period. Participants belonging to the highest quartile of the Lifelines Diet Score (LLDS) compared to those in the lowest quartile exhibited a reduced incidence and mortality of lung cancer including small cell lung cancer. It is noteworthy that pre-defined potential effect modifiers such as age, sex, smoking status, genetic factors, pre-existing lung conditions, aspirin use and body mass index, did not impact the association between LLDS and the incidence and mortality of lung cancer. These findings highlight the need for all to maintain a healthy diet in reducing the risk of lung cancer and its consequences, particularly for adults aged 55 or above.

Building on the evidence supporting dietary influences on lung cancer risk, the consumption of raw white garlic has been promoted for its health benefits in different cultures (7). The findings from 12 clinical trials, and 10 observational studies involving only Asian populations have been reported (Fejes et al.). Although there is some evidence supporting improved lipid profiles, blood pressure regulation, fibrinolytic activity, anti-oxidant status, glucose metabolism and apoptosis following white garlic consumption, none of these were directly associated with lowered cancer risks and mortality. The observational studies reported that raw garlic consumption was associated with reduced incidence of liver, esophageal and lung cancers. Overall, raw white garlic consumption may regulate cellular processes that reduce tumor development. However, the studies reviewed were heterogenous in many aspects and multiple separate trials involving different types of cancer patients are warranted to validate this hypothesis. In addition to dietary patterns and specific food components, the intake of common beverages has also been investigated for its potential role in cancer prevention. Intake of caffeinated beverages such as coffee and tea may also influence the risks of malignancies such as glioma. Pan et al. conducted a meta-analysis that suggested an inverse correlation between drinking more than 2.5 cups of tea per day and a reduced risk of glioma while coffee intake failed to show such a relationship.

Blindness and vision impairment affects at least 2.2 billion people where half of these cases could have been prevented (8). One of the leading causes of this chronic condition is age-related macular degeneration (AMD) (9). This disease is characterized by a progressive loss of cells in the central region of the retina. It is known that patients with AMD tend to experience an imbalanced fatty acid profile compared to healthy individuals (10). Having analyzed National Health and Nutrition Examination Survey (NHANES) data from 2005 to 2008, Xu et al. reported reduced levels of monounsaturated and polyunsaturated fatty acids in people with AMD. A daily intake of fatty acids that include the omega-3 fatty acid, docosapentaenoic acid (DPA), was also lower in the AMD group; thus suggesting the benefits of DPA intake in AMD. The NHANES data also revealed the significance of sustaining a healthy oxidative balance to preserve visual function; this can be achieved by engaging in favorable lifestyle practices like enhanced physical activity, non-smoking habits and reduced consumption of alcohol (Li et al.). These studies support the impact that dietary and lifestyle interventions have on maintaining eye health and vision.

Conditions affecting the musculoskeletal system are often incurable and most treatments focus on managing symptoms rather than impeding the progression of muscle degradation and loss of function (11). Therefore, it is important to preserve muscle mass, strength and function. An example of a chronic skeletal muscle disorder is sarcopenia and it typically affects adults, compromising their quality of life over time. It has long been substantiated that increased physical performance such as exercise training can help people with sarcopenia and more recently, protein-based supplements including β-hydroxy-β-methylbutyrate (HMB) have been under investigation for their potential benefits in patients and the general population (12). A meta-analysis conducted by Feng and their colleagues have showed that HMB supplementation may enhance physical performance (i.e., gait speed) in patients with sarcopenia compared to the placebo group who had undergone an exercise regimen. However, combining exercise with HMB supplementation did not alter skeletal muscle index in people with sarcopenia (Feng et al.). In inherited musculoskeletal diseases including various types of hereditary ataxias, holistic treatment strategies integrating nutraceuticals (e.g., Vitamin B1, Vitamin D, trehalose, and polyunsaturated fatty acids) and rehabilitation programs that promote physical activity (e.g., walking, yoga, cycling, treadmill activities, transcranial magnetic stimulation with physiotherapy, trunk training, intensive balance training, and core stability exercise) with current methods of symptomatic management is recommended for patients as soon as they are clinically diagnosed (Yang et al.).

Beyond neurological and rehabilitative interventions, metabolic and gastrointestinal conditions also represent significant public health concerns. Gallstones also known as ‘cholelithiasis' is a common gastrointestinal tract disorder affecting up to 20% of people worldwide (13). One common aetiological factor is a high intake of foods rich in carbohydrates and saturated fats; and the development of gallstones is often asymptomatic until advanced stages, causing abdominal pain and an increased risk of infections and cancer of the gall blader (14, 15). Using the data from NHANES, oxidative balance scores (OBS) incorporating dietary and lifestyle variables were analyzed (Yang et al.). Lower OBS corresponding to higher levels of oxidative stress in the body conferred a higher risk of developing cholelithiasis; and this relationship reported diabetes and cardiovascular disease status as covariates. A large-scale prospective cohort study analyzed various dietary patterns: Mediterranean Diet Score (MED), alternate Mediterranean Diet Score (aMED), Plant-based Diet Index (PDI), healthy Plant-based Diet Index (hPDI), unhealthy Plant-based Diet Index (uPDI), Healthy Eating Index 2015 (HEI-2015) and EAT-Lancet Score (Jin et al.). It was determined that following the aMED emphasizing on whole foods, healthy fats and plant-based ingredients, and adhering to HEI-2015 measurements by limiting intakes of both added sugars and saturated fats to less than 10% of one's total energy source, lowered the risk of developing gallstones. The results from these studies underscore the importance of consuming food rich in anti-oxidants, fiber and unsaturated fats in maintaining a healthy gastrointestinal system comprising the gall bladder.

Building on the evidence that diet quality and lifestyle factors can influence, recent research has also explored the impact of metabolic and dietary factors on musculoskeletal health, particularly osteoarthritis (OA). OA is the most prevalent musculoskeletal disorder, causing joint pain, functional limitations, and reduced quality of life in older adults (16, 17). Using NHANES data from 3,779 adults aged 65 years and older, Huang et al. found that higher visceral fat accumulation, measured by the Visceral Adiposity Index (VAI), was strongly associated with increased OA prevalence, with participants in the highest VAI quartile exhibiting more than double the risk compared to the lowest quartile. In parallel, Ji et al. employing logistic regression and Mendelian randomization approaches demonstrated that higher caffeine intake, especially coffee consumption exceeding 95 mg/day, was linked to elevated risk of OA, with consistent findings across multiple analytical methods.

Extending the focus from osteoarthritis to broader musculoskeletal health, recent research has explored the influence of diet, body composition, and oxidative balance on bone mineral density (BMD) and osteoporosis (OP). A cross-sectional study carried out by Austin et al. of 240 adults aged 30–75 compared various plant-based diets (vegan, lacto-vegetarian, pesco-vegetarian, semi-vegetarian) with a regular meat diet and found no significant differences in whole-body BMD or body composition, except for lower lean mass and T-scores in lacto-ovo vegetarians, highlighting that appropriately planned plant-based diets can maintain bone health when calcium and protein intake are adequate. Ding et al. used data from NHANES englobing 9,295 adults and observed that higher Body Roundness Index (BRI) was inversely associated with total BMD, with each unit increase linked to a measurable reduction in BMD and a pronounced effect beyond an inflection point of 9.52, suggesting that central obesity may elevate the risk of osteoporosis. In line with these findings, in 2,862 postmenopausal women from NHANES, higher Comprehensive Dietary Antioxidant Index (CDAI) scores were positively associated with femoral bone mineral density and significantly linked to a lower risk of osteoporosis, with non-linear relationships observed across age groups (Sun et al.). Complementing this, analysis of 776 adults aged 50 years and older demonstrated that higher oxidative balance scores (OBS), reflecting diets rich in antioxidants and healthy lifestyle practices, were inversely associated with all-cause mortality among OP patients, underscoring the protective role of antioxidant-rich nutrition (Ding et al.). Beyond dietary factors, body composition metrics such as the Body Roundness Index (BRI) were also shown to impact long-term outcomes, with higher BRI levels associated with improved survival in osteoporotic individuals, demonstrating an “L”-shaped inverse relationship with mortality (Ding et al.).

Obesity measured by the BRI has also been linked to other health outcomes (18–20). Higher BRI was associated with an increased risk of female infertility in NHANES women aged 18–45, with participants in the highest quartile exhibiting more than double the odds compared to the lowest quartile and a non-linear relationship observed (Li et al.). In addition, obesity can influence sleep, which is a critical determinant of overall health, affecting cognitive function, metabolic regulation, cardiovascular and cancer risk (21–23). In elderly Chinese adults, higher BMI, waist circumference, and waist-to-height ratio were associated with better sleep quality, whereas underweight individuals were more likely to experience poor sleep (Liang et al.). Complementing these observational findings, the dietary supplement LTC-022, containing Lactium and L-theanine, improved total sleep time, sleep efficiency, and bedtime regularity, alongside beneficial changes in gut microbiota composition, suggesting a mechanistic link between diet, microbiome, and sleep regulation (Lim et al.).

By preventing the onset or progression of chronic illness and promoting healthy aging through simple, self-managed intervention, such as maintaining a balanced diet and engaging in regular physical activity, we can significantly reduce the global economic and social burden of disability. Empowering individuals through health literacy programs, and fostering supportive environments via government, community, and medical organization-led advocacy, will reinforce positive lifestyle choices and enable people to enjoy a good quality of life as they age.

## References

[1] Gianfredi V Nucci D Pennisi F Maggi S Veronese N Soysal P. Aging, longevity, and healthy aging: the public health approach. Aging Clin Exp Res. (2025) 37:125. doi: 10.1007/s40520-025-03021-8 40244306 PMC12006278

[2] Bardach SH Schoenberg NE. The role of primary care providers in encouraging older patients to change their lifestyle behaviors. Clin Gerontol. (2018) 41:326–34. doi: 10.1080/07317115.2017.1376029 29221431 PMC5893434

[3] Amiri S Mahmood N Junaidi S Khan MA. Lifestyle interventions improving health-related quality of life: a systematic review and meta-analysis of randomized control trials. J Educ Health Promot. (2024) 13:193. doi: 10.4103/jehp.jehp_1156_23 39268447 PMC11392327

[4] González-González E Requena C. Self-care interventions of community-dwelling older adults: a systematic review and meta-analysis. Front Public Health. (2023) 11:1254172. doi: 10.3389/fpubh.2023.1254172 37876713 PMC10593480

[5] World Health Organisation. Cancer (2025). Available online at: https://www.who.int/news-room/fact-sheets/detail/cancer (Accessed February 3, 2025).

[6] Feng M Wang F Bao M Zhu L. Environmental risk factors, protective factors and lifestyles for lung cancer: an umbrella review. Front Public Health. (2025) 13:1623840. doi: 10.3389/fpubh.2025.1623840 40766027 PMC12321780

[7] El-Saadony MT Saad AM Korma SA Salem HM Abd El-Mageed TA Alkafaas SS . Garlic bioactive substances and their therapeutic applications for improving human health: a comprehensive review. Front Immunol. (2024) 15:1277074. doi: 10.3389/fimmu.2024.1277074 38915405 PMC11194342

[8] World Health Organization. Blindness and Vision Impairment (2023). Available online at: https://www.who.int/news-room/fact-sheets/detail/blindness-and-visual-impairmenthttps://www.who.int/news-room/fact-sheets/detail/blindness-and-visual-impairment (Accessed October 12, 2023).

[9] GBD 2019 Blindness and Vision Impairment Collaborators Vision Vision Loss Expert Group of the Global Burden of Disease Study. Causes of blindness and vision impairment in 2020 and trends over 30 years, and prevalence of avoidable blindness in relation to VISION 2020: the Right to Sight: an analysis for the Global Burden of Disease Study. Lancet Glob Health. (2021) 9:e144–e60. doi: 10.1016/S2214-109X(20)30489-7 33275949 PMC7820391

[10] Kwa F Dulull N Roessner U Dias D Rupasinghe T. Lipidomics reveal the protective effects of a vegetable-derived isothiocyanate against retinal degeneration. F1000Research. (2020) 8:19598. doi: 10.12688/f1000research.19598.3 33145006 PMC7590896

[11] Lilley T Camera DM Kwa FAA. Repairing muscle with broccoli-derived sulforaphane: a preclinical evaluation for the treatment of mitochondrial myopathies. Drug Discov Today. (2025) 30:104283. doi: 10.1016/j.drudis.2024.104283 39736463

[12] Holeček M. Beta-hydroxy-beta-methylbutyrate supplementation and skeletal muscle in healthy and muscle-wasting conditions. J Cachexia Sarcopenia Muscle. (2017) 8:529–41. doi: 10.1002/jcsm.12208 28493406 PMC5566641

[13] Portincasa P Di Ciaula A Bonfrate L Stella A Garruti G Lamont JT. Metabolic dysfunction-associated gallstone disease: expecting more from critical care manifestations. Intern Emerg Med. (2023) 18:1897–918. doi: 10.1007/s11739-023-03355-z 37455265 PMC10543156

[14] Kotrotsios A Tasis N Angelis S Apostolopoulos AP Vlasis K Papadopoulos V . Dietary intake and cholelithiasis: a review. J Long Term Eff Med Implants. (2019) 29:317–26. doi: 10.1615/JLongTermEffMedImplants.2020034732 32749137

[15] Huang D Joo H Song N Cho S Kim W Shin A. Association between gallstones and the risk of biliary tract cancer: a systematic review and meta-analysis. Epidemiol Health. (2021) 43:e2021011. doi: 10.4178/epih.e2021011 33541011 PMC8060519

[16] Katz JN Arant KR Loeser RF. Diagnosis and treatment of hip and knee osteoarthritis: a review. JAMA. (2021) 325:568–78. doi: 10.1001/jama.2020.22171 33560326 PMC8225295

[17] Yan H Guo J Zhou W Dong C Liu J. Health-related quality of life in osteoarthritis patients: a systematic review and meta-analysis. Psychol Health Med. (2022) 27:1859–74. doi: 10.1080/13548506.2021.1971725 34465255

[18] Zhang X Ma N Lin Q Chen K Zheng F Wu J . Body roundness index and all-cause mortality among US adults. JAMA Netw Open. (2024) 7:e2415051. doi: 10.1001/jamanetworkopen.2024.15051 38837158 PMC11154161

[19] Yang M Liu J Shen Q Chen H Liu Y Wang N . Body roundness index trajectories and the incidence of cardiovascular disease: evidence from the China health and retirement longitudinal study. J Am Heart Assoc. (2024) 13:e034768. doi: 10.1161/JAHA.124.034768 39319466 PMC11681446

[20] Chen Z Cheang I Zhu X Qu Q Chen S Xing Y . Associations of body roundness index with cardiovascular disease and mortality among patients with metabolic syndrome. Diabet Obes Metab. (2025) 27:3285–98. doi: 10.1111/dom.16346 40145489

[21] Korostovtseva L Bochkarev M Sviryaev Y. Sleep and Cardiovascular Risk. Sleep Med Clin. (2021) 16:485–97. doi: 10.1016/j.jsmc.2021.05.001 34325825

[22] Leng Y Knutson K Carnethon MR Yaffe K. Association between sleep quantity and quality in early adulthood with cognitive function in midlife. Neurology. (2024) 102:e208056. doi: 10.1212/WNL.0000000000208056 38170947 PMC10870739

[23] Lozano-Lorca M Olmedo-Requena R Vega-Galindo MV Vázquez-Alonso F Jiménez-Pacheco A Salcedo-Bellido I . Night shift work, chronotype, sleep duration, and prostate cancer risk: CAPLIFE study. Int J Environ Res Public Health. (2020) 17:6300. doi: 10.3390/ijerph17176300 32872503 PMC7503878

